# Psychosocial Health Following Non-Invasive Brain Stimulation of Oculomotor Regions

**DOI:** 10.3390/bs16060897

**Published:** 2026-06-02

**Authors:** Suraj Upadhyaya

**Affiliations:** Chicago College of Optometry, Midwestern University, Downers Grove, IL 60515, USA; supadh@midwestern.edu

**Keywords:** tDCS, frontal eye field, cerebellum, PANAS, affective modulation, oculomotor neuroscience, brain stimulation

## Abstract

Emotional well-being is an important consideration when applying non-invasive brain stimulation to regions involved in visual and oculomotor control. This study assessed affective changes following transcranial direct current stimulation (tDCS) targeting the frontal eye field (FEF) and cerebellum using the Positive and Negative Affect Schedule (PANAS). Twenty-five healthy adults completed PANAS assessments after anodal and sham tDCS delivered in separate sessions. A repeated-measures general linear model evaluated stimulation type (anodal vs. sham), stimulation site (FEF vs. cerebellum), and time (pre- vs. post-stimulation). For positive affect, no significant interaction or main effects were observed across type, site, or time (all *p* > 0.05), with nonsignificant effects of site (*p* = 0.07) and stimulation type (*p* = 0.97). For negative affect, neither type (*p* = 0.11) nor site (*p* = 0.48) showed significant main effects; however, a significant three-way interaction among time, site, and type emerged (*p* = 0.01). Across all stimulation conditions, both positive and negative affect decreased post-stimulation. These findings indicate that tDCS targeting oculomotor regions does not produce adverse affective outcomes and is emotionally safe for experimental use. Future work should use extended stimulation protocols in clinical populations to further examine tDCS-related emotional modulation.

## 1. Introduction

Emotions play a fundamental role in shaping human behavior, influencing attention, memory, decision-making, and action selection, as shown in extensive neuroscientific reviews (Dolan, 2002; Levine, 2022). They guide how individuals perceive their environment, prioritize information, and regulate responses to salient stimuli, reflecting the close integration of emotional and cognitive processes across the brain (Pessoa, 2017; Palomero-Gallagher & Amunts, 2022). Emotional states can bias motor responses, alter cognitive performance, and modulate attention, illustrating that affective, sensory, motor, and executive systems operate through interconnected neural networks.

While traditional models of emotion emphasize the roles of the prefrontal cortex and limbic structures, contemporary research reveals that emotional regulation relies on distributed networks involving cortical, subcortical, and cerebellar structures (Casey et al., 2019; Pierce & Péron, 2020). These circuits coordinate the appraisal of emotional stimuli, autonomic responses, and motor expressions of emotion, reflecting large-scale network interactions rather than isolated regional functions (Pessoa, 2017; Smith et al., 2021). Within this broader framework, two regions—the cerebellum and the frontal eye field (FEF)—have emerged as important nodes in the interaction between emotional and sensorimotor systems.

The cerebellum, historically viewed as a motor structure, is now known to contribute to emotional processing through its extensive connections with the prefrontal cortex, limbic system, and autonomic centers (Clausi et al., 2017; Ciapponi et al., 2023). Cerebellar dysfunction is associated with affective disturbances, including depression and emotional dysregulation syndromes (Frazier et al., 2022). Importantly, cerebellar stimulation—such as transcranial direct current stimulation (tDCS)—has been shown to produce measurable changes in brain activity and behavior. Studies report enhanced mood states, altered perception of negative emotions, and modulation of cognitive–affective integration following cerebellar tDCS (Ferrucci & Priori, 2014; Newstead et al., 2018). These findings highlight the cerebellum’s role as an integrative hub supporting both motor coordination and emotional regulation.

The FEF plays a critical role in voluntary eye movements, attentional allocation, and executive control. As a component of the oculomotor network, the FEF generates saccadic eye movements, stabilizes gaze, and shapes how visual information is prioritized. Its connections with prefrontal and limbic regions position it as a pathway through which attention and emotion interact (Subramaniam et al., 2023). Evidence shows that frontal stimulation can reduce emotional reactivity to negative stimuli, likely by enhancing prefrontal inhibitory control over limbic structures such as the amygdala (Clarke et al., 2021). Although primarily studied in visuomotor contexts, the FEF may therefore influence emotional processing through top-down regulatory mechanisms.

This study evaluated affective changes following transcranial direct current stimulation (tDCS) of the FEF and cerebellum using the Positive and Negative Affect Schedule (PANAS). The PANAS, developed by Watson and colleagues, is a widely validated self-report measure of transient and dispositional affect (Watson et al., 1988). It demonstrates strong reliability and construct validity across clinical and nonclinical samples (Crawford & Henry, 2004) and correlates well with longer mood inventories such as the Hopkins Symptom Checklist and Beck Depression Inventory. Its applicability across developmental groups (Hughes & Kendall, 2009; Veronese & Pepe, 2017) and diverse cultural settings (Díaz-García et al., 2020; Hovmand et al., 2023; L. Song et al., 2024; X. Song et al., 2024; Wang, 2024) has made it a standard tool for capturing subtle mood fluctuations in psychological and biomedical research.

Transcranial Direct Current Stimulation is a non-invasive neuromodulation technique that alters cortical excitability through weak anodal (excitatory) or cathodal (inhibitory) currents (Woods et al., 2016). Its therapeutic potential has been demonstrated across neurological, psychiatric, and cognitive disorders (Kuo et al., 2017; Kim et al., 2018; Camacho-Conde et al., 2023), including Post-Traumatic Stress Disorder (PTSD), generalized anxiety disorder, and depression (Salehinejad et al., 2017; Ahmadizadeh et al., 2019). Beyond mood disorders, tDCS has been used to enhance motor learning, modulate ocular motor behaviors, and reduce pain (Buch et al., 2017; Gan et al., 2022; Moshfeghinia et al., 2023). Given its capacity to influence broad cortical and subcortical networks, unintended spillover effects on emotional processing are plausible. Yet, emotional outcomes of tDCS—especially during stimulation of nontraditional affect-related regions—remain understudied, and tools such as PANAS are rarely employed in these contexts.

Although most tDCS research targets the prefrontal cortex, cerebellar stimulation has gained attention for its potential in treating motor and neurodegenerative disorders, including cerebral palsy and Parkinson’s disease (Villalta Santos et al., 2019; Pisano et al., 2024). The cerebellum’s dense architecture and extensive connections with cortical and limbic circuits enable it to influence affective processing. It has been implicated in the modulation of negative emotions, and cerebellar tDCS has been shown to enhance mood states (Ferrucci & Priori, 2014; Newstead et al., 2018).

The frontal eye fields (FEF), classically associated with voluntary eye movements and visuospatial attention, have increasingly been examined as a target for visual and oculomotor rehabilitation. Functional MRI studies demonstrate robust FEF engagement during vision therapy tasks (Alvarez et al., 2010), and recent evidence indicates that anodal tDCS applied over the left FEF can reduce symptoms of convergence insufficiency (Max et al., 2024) and modulate fixation behavior (Sharma & Upadhyaya, 2026), positioning the FEF as a promising intervention target for visual function. In contrast, the cerebellum has been increasingly implicated in broader integrative processes supporting emotional and cognitive regulation, with contemporary models suggesting that its contributions to affect involve sensorimotor–affective integration and predictive modeling of emotional responses across contexts rather than direct executive control (Rudolph et al., 2023).

Directly comparing the FEF and cerebellum is therefore essential for differentiating their roles in affective modulation. While FEF stimulation may influence emotion primarily through attentional gating and executive pathways, cerebellar stimulation appears to engage distributed networks integrating somatic, cognitive, and autonomic components of emotion. Understanding these distinct pathways has important implications for targeted neuromodulation therapies tailored to specific emotional dysfunctions—whether driven by attentional biases, executive deficits, or failures in sensorimotor-emotional integration. This study investigates whether tDCS targeting the FEF or cerebellum—each tested under anodal and sham conditions—elicits measurable changes in affective states in healthy adults. By comparing two functionally distinct regions, this study aims to clarify their respective contributions to emotional modulation and inform future neuromodulation approaches for visual and affective disorders.

## 2. Materials and Methods

This cross-sectional prospective study was conducted at the Chicago College of Optometry in Downers Grove, IL, USA, and received approval from the Institutional Review Board of Midwestern University (IRB no: 21064). All study procedures adhered to the principles of the Declaration of Helsinki. Written informed consent was obtained from each participant following a detailed explanation of the study protocol. Data collection occurred between 11 October 2021 and 10 October 2023.

Participants were eligible if they were 18 to 65 years old, regardless of gender, race, or ethnicity, and had normal or corrected-to-normal vision. Individuals were excluded if they had a history of neurological or psychiatric disorders, epilepsy, or were undergoing treatment or taking medications for such conditions. Additional exclusion criteria included the presence of metallic implants in the head or heart (except titanium) and pregnancy.

Brain stimulation was delivered using a DC Stimulator MR (NeuroConn, Brainbox Ltd., Cardiff, UK). To ensure accurate electrode placement, a neuronavigation system (BrainSight, Brainbox Ltd., Cardiff, UK) was used in combination with a virtual brain template to individually target either the left frontal eye field (FEF) or the cerebellum. The left FEF was localized using well-established Montreal Neurological Institute coordinates (X = −31, Y = −3, Z = 57) (Heinen et al., 2014). Electrodes were positioned in saline-soaked sponges and secured with rubber straps.

Cerebellar tDCS was administered using a constant direct-current stimulator with rectangular saline-soaked synthetic sponge electrodes (5 × 5 cm). The active electrode was centered on the midline, 2 cm below the inion, with its lateral edges approximately 1 cm medial to the mastoid processes. The reference electrode was placed over the right arm. This extracephalic montage, employed in previous cerebellar tDCS studies, minimizes potential confounds arising from two oppositely polarized electrodes placed on the scalp (Ferrucci et al., 2015).

Active stimulation consisted of a 2.0 mA current applied over a 25 cm^2^ area for 25 min, with 20 s ramp-up and ramp-down periods. Sham stimulation involved an initial 20 s ramp-up to a nominal peak of 0 mA, followed immediately by a ramp-down, after which the current remained at zero for the rest of the session. The decision to use 2.0 mA was based on evidence from Ehrhardt et al. (Ehrhardt et al., 2021) showing that increased tDCS intensities yield more robust and reliable behavioral effects. Importantly, this level remains within the safety parameters defined by Bikson et al. (Bikson et al., 2016).

Participants were randomly assigned using an online randomization generator to receive either active anodal tDCS or sham stimulation targeting the frontal eye fields (FEF) or cerebellum. Each participant completed four stimulation sessions scheduled 48–72 h apart. The sessions corresponded to four conditions (FEF-anodal, FEF-sham, cerebellum-anodal, cerebellum-sham), with their order counterbalanced across participants using a set of predefined sequences generated by the randomization tool. These sequences were designed to ensure balanced representation of stimulation site and condition at each session, preventing disproportionate exposure to any condition across visits. Participants were blinded to the stimulation condition during each session. This counterbalanced within-subject design minimized systematic order effects while maintaining equal exposure to all experimental conditions. Based on the distinct neurobiological roles of the frontal eye fields (FEF) and the cerebellum, we formulated a priori hypotheses regarding stimulation- and site-dependent modulation of affect. We hypothesized that (1) anodal stimulation would be associated with time-dependent changes in PANAS positive and/or negative affect scores relative to sham stimulation, and (2) these changes would differ as a function of stimulation site (FEF vs. cerebellum), reflecting their putative roles in attentional/executive gating and cognitive–affective integration, respectively. Accordingly, we first examined the main effects of stimulation type, site, and timing, and then conducted inferential tests focused on interaction effects within a repeated-measures general linear model (GLM), including Site × Stimulation × Time interactions, analyzed separately for positive and negative affect composite scores.

### 2.1. PANAS Survey

The Positive and Negative Affect Schedule (PANAS) was used to assess changes in emotional state associated with stimulation. PANAS is a standardized 20-item self-report measure comprising two 10-item subscales that assess positive affect (PA) and negative affect (NA) (Watson et al., 1988). Participants rated each item on a 5-point Likert scale ranging from 1 (“very slightly or not at all”) to 5 (“extremely”), and subscale scores were calculated by summing responses to the corresponding 10 items, yielding a score range of 10 to 50 for both PA and NA.

Participants completed the PANAS at two time points during each session: approximately 5 min prior to stimulation onset and within 5 min following the end of stimulation. The post-stimulation assessment was administered immediately after stimulation to capture acute effects while minimizing delay-related confounds. Each participant completed both pre- and post-session assessments for all four stimulation conditions (FEF-anodal, FEF-sham, cerebellum-anodal, cerebellum-sham), enabling within-subject comparisons of affective responses across stimulation sites and conditions. Standardized instructions were provided at each session, and participants completed the questionnaire independently without researcher input or feedback.

### 2.2. Data Acquisition and Analysis

Emotional response data were quantified using PANAS scores to evaluate changes associated with stimulation type and targeted brain region. Analyses examined differences in positive and negative affect following anodal versus sham stimulation, providing insights into the potential emotional effects of anodal tDCS in normal populations. Cumulative scores for positive and negative affect were calculated for each survey and compared before and after stimulation within each condition.

A repeated-measures general linear model was used to examine the effects of stimulation type (anodal vs. sham), stimulation site (FEF vs. cerebellum), and time point (before vs. after stimulation). All statistical analyses were conducted using SPSS 16.0, with significance set at α = 0.05.

## 3. Results

A total of 25 participants were included in this study. The mean age was 25.76 ± 2.33 years, with 55.8% identifying as female and 44.2% as male. Table 1 presents descriptive statistics—including mean, standard deviation, median, range, skewness, and kurtosis for all measured variables. To examine whether transcranial direct current stimulation (tDCS) influenced emotional states, we first compared anodal and sham stimulation on positive and negative emotion scores by combining both sites.

### 3.1. Combine Analysis

**Figure 1:**
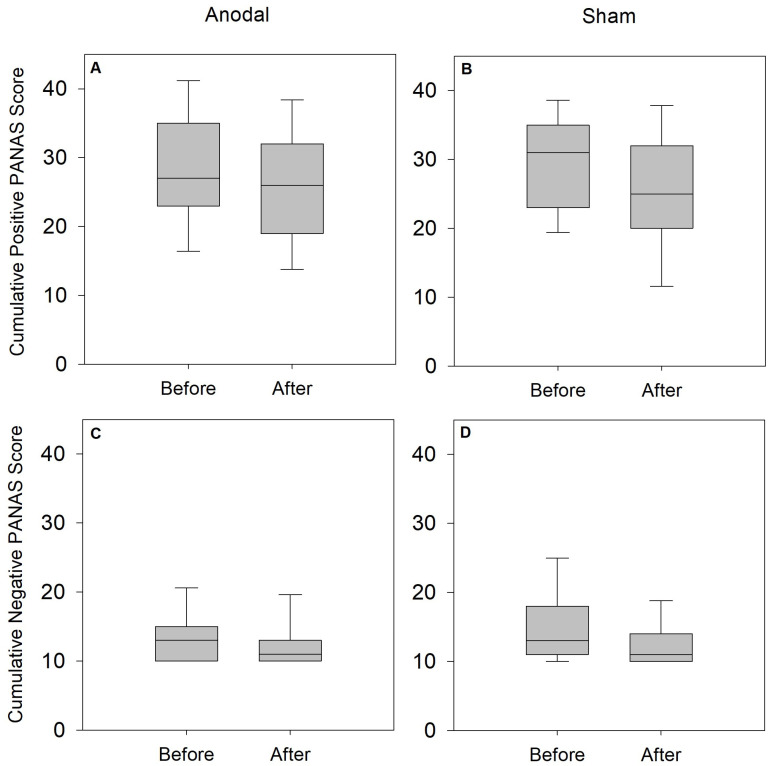
Figure 1 presents box plots illustrating Positive and Negative Affect Schedule (PANAS) scores before and after transcranial direct current stimulation (tDCS). Each box represents the interquartile range (25th–75th percentiles), with the median shown by a solid black line. Panel (**A**) displays positive affect scores before and after anodal tDCS, whereas Panel (**B**) shows positive affect scores before and after sham stimulation. Panel (**C**) depicts negative affect scores before and after anodal tDCS, and Panel (**D**) shows negative affect scores before and after sham stimulation. Abbreviations: tDCS = transcranial direct current stimulation; PANAS = Positive and Negative Affect Schedule.

Descriptively, Figure 1 illustrates overall pre- to post-stimulation changes in PANAS outcomes collapsed across stimulation sites, highlighting trends between anodal and sham conditions. Consistent with these pooled observations, inferential analyses using a repeated-measures general linear model (GLM) were performed with Site, Stimulation Type, and Time as within-subject factors, separately for positive and negative affect. The GLM results (reported below) provide the formal statistical evaluation of these effects, while Figure 1 serves as a summary visualization. Site-specific patterns are presented in subsequent figures to complement these pooled results.

Across all participants, the cumulative positive PANAS score before anodal tDCS had a median of 27 (Interquartile Range = 12), which decreased to 26 (13) after stimulation. Before sham tDCS, the median positive score was 31 (12), declining to 25 (12) post-stimulation. For negative affect, the median score before anodal tDCS was 13 (5), decreasing to 11 (3) after stimulation. Before sham tDCS, the median negative score was 13 (7), which decreased to 11 (4) post-stimulation as shown in Figure 1A–D.

To aid interpretation, pre- to post-stimulation change scores (Δ PANAS) were computed descriptively as the difference between post- and pre-stimulation means. Anodal stimulation over both the FEF and cerebellum was associated with small numerical reductions in positive and negative affect scores. For FEF stimulation, mean Δ scores were −1.73 for positive affect and −1.69 for negative affect. For cerebellar stimulation, mean Δ scores were −4.12 for positive affect and −0.47 for negative affect. These descriptive results indicate modest changes in affect following stimulation, with no marked qualitative differences in the overall direction of effects across stimulation sites.

The repeated-measures GLM revealed that the Stimulation × Time interaction was not statistically significant for either positive or negative affect, indicating that pre–post changes did not differ reliably between anodal and sham conditions when averaged across stimulation sites. These findings suggest no overall differential effect of stimulation over time at the pooled level for either outcome.

In contrast, a significant Site × Stimulation × Time interaction was observed for negative affect, indicating that changes over time varied as a function of stimulation condition in a site-dependent manner. This pattern suggests that stimulation effects are not uniform across sites and are better understood within specific site contexts rather than through pooled estimates. To clarify this interaction, follow-up simple-effects analyses are presented below, focusing on the direction and magnitude of pre–post changes within individual sites without overinterpreting non-significant pooled effects.

### 3.2. Frontal Eye Field (FEF) Stimulation

**Figure 2:**
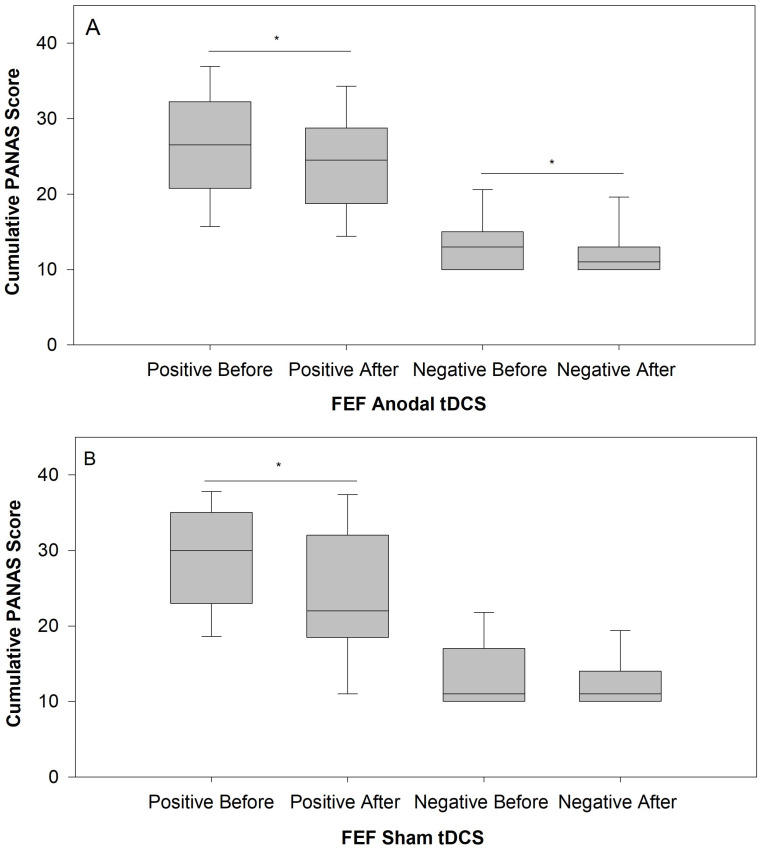
Figure 2 shows box plots of combined positive and negative PANAS affect scores measured before and after tDCS targeting the frontal eye field (FEF). Each box represents the interquartile range (25th–75th percentiles), with the median indicated by a solid black line. Asterisks (*) denote statistically significant changes at *p* < 0.01. Panel (**A**) presents cumulative affect scores before and after anodal FEF stimulation, while Panel (**B**) presents cumulative affect scores before and after sham FEF stimulation. Abbreviations: tDCS = transcranial direct current stimulation; FEF = frontal eye field; PANAS = Positive and Negative Affect Schedule.

In the FEF group, the median positive PANAS score before anodal tDCS was 26.5 (11.5), decreasing to 24.5 (10) after stimulation. Before sham stimulation, the median was 30 (12) and declined to 22 (13.5) post-stimulation. For negative affect, the median score before anodal tDCS was 12 (5), decreasing to 11 (1.25) after stimulation. Before sham stimulation, the median was 11 (7) and remained 11 (4) afterward. Descriptively, positive emotion scores declined following both anodal and sham stimulation. Negative emotion scores showed a modest reduction after anodal stimulation, whereas sham stimulation produced no noticeable change (Figure 2A,B).

### 3.3. Cerebellar Stimulation

**Figure 3:**
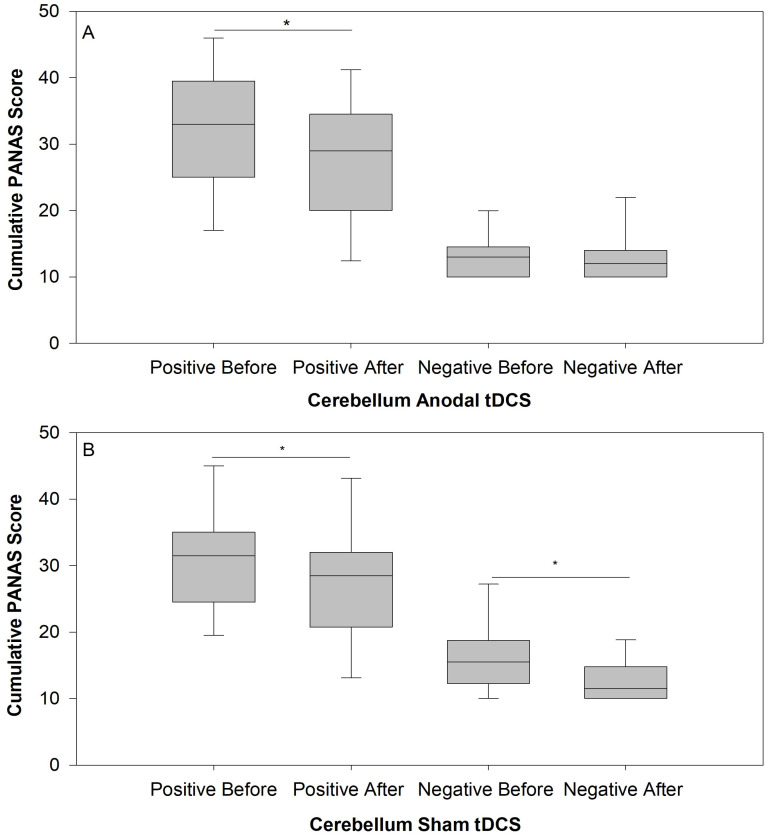
Figure 3 displays box plots of combined positive and negative PANAS affect scores before and after cerebellar tDCS. Each box shows the interquartile range (25th–75th percentiles), with a solid black line indicating the median. Asterisks (*) mark statistically significant differences at *p* < 0.01. Panel (**A**) illustrates cumulative affect scores before and after anodal cerebellar stimulation, and Panel (**B**) shows cumulative affect scores before and after sham cerebellar stimulation. Abbreviations: tDCS = transcranial direct current stimulation; PANAS = Positive and Negative Affect Schedule.

In the cerebellar group, the median positive PANAS score before anodal tDCS was 33 (14.5), decreasing to 29 (14.5) after stimulation. Before sham stimulation, the median was 31.5 (11.5), decreasing to 28.5 (11.75) post-stimulation. For negative affect, the median score before anodal stimulation was 13 (4.5), slightly decreasing to 12 (4) afterward. Before sham stimulation, the median was 15.5 (6.5), decreasing to 11.5 (4.75) after stimulation. Overall, positive emotions decreased following both anodal and sham stimulation. Negative emotions showed no change after anodal stimulation but decreased following sham stimulation (Figure 3A,B).

### 3.4. Statistical Analysis (Repeated-Measures General Linear Model)

A repeated-measures general linear model revealed a significant reduction in both positive and negative emotional scores after stimulation, regardless of stimulation type (anodal vs. sham) or site (FEF vs. cerebellar). For positive emotion scores, there was a significant main effect of time F(1) = 40.12, *p* < 0.001, indicating reduced scores following stimulation. No significant main effects of stimulation type or site emerged, and no two-way or three-way interactions were significant. Pairwise comparisons confirmed significantly lower post-stimulation scores after adjustment for multiple comparisons. For negative emotion scores, a significant main effect of time was also observed F(1) = 21.49, *p* < 0.001, indicating decreases across both stimulation conditions. Although two-way interactions were not significant, a significant three-way interaction among stimulation type × site × time was found F(1) = 7.05, *p* = 0.01. Post hoc analyses revealed that within the FEF site, pre-stimulation negative emotion scores differed significantly between anodal and sham conditions. Overall, these findings suggest that tDCS—whether anodal or sham—is associated with reductions in emotional intensity.

## 4. Discussion

This study examined the emotional effects of anodal transcranial direct current stimulation (tDCS) applied to the FEF and cerebellum using the Positive and Negative Affect Schedule (PANAS). Emotional states were assessed before and after active and sham stimulation sessions. The primary findings revealed that both anodal and sham stimulation produced changes in positive and negative affect across all stimulation sites. Positive affect declined in both conditions, though the reduction was greater following sham stimulation. Negative affect decreased in both groups as well, with a more pronounced reduction under anodal stimulation. Together, these findings suggest that anodal tDCS may subtly protect against mood deterioration and may enhance reductions in negative affect relative to sham.

When examining stimulation sites individually, anodal FEF stimulation produced a statistically significant reduction in negative affect compared to sham, while changes in positive affect were significant in both sham and anodal conditions. In contrast, sham stimulation yielded a significant decrease in positive affect, suggesting that active stimulation may help preserve positive mood. For cerebellar stimulation, positive affect decreased under both anodal and sham conditions, but the decline was larger under sham, reflecting a consistent protective trend with anodal stimulation. Negative affect decreased for both conditions, though statistical significance was achieved only with sham stimulation. Overall, these results indicate that anodal tDCS over the FEF and cerebellum is emotionally well tolerated and may offer mild mood-stabilizing effects.

These findings contribute to a growing body of literature supporting the neuromodulatory potential of noninvasive brain stimulation. Brain stimulation has gained traction as an adjunctive approach in treating psychiatric and neurological conditions (Camacho-Conde et al., 2023). Although repetitive transcranial magnetic stimulation (TMS) remains widely used, tDCS is emerging as an attractive alternative due to its low cost, portability, and minimal risk profile. The rapid increase in tDCS clinical trials highlights its potential for broader application, including integration with closed-loop systems and use in populations for whom TMS or deep brain stimulation may be less accessible or reserved for more severe cases.

Most mood-related tDCS studies have focused on the prefrontal cortex. However, accumulating evidence points to the cerebellum as an important substrate for affective processing. Newstead et al. (2018) found that stimulation of both the prefrontal cortex and the cerebellum significantly improved mood in healthy participants using the profile of mood states (POMS) visual analogue scale, whereas no improvement occurred in the sham condition. Direct comparison with our findings is limited due to differences in measurement scales, as POMS assesses more sustained mood states while PANAS captures momentary positive and negative affect. Similarly, Salehinejad et al. (2017) demonstrated that dorsolateral prefrontal cortex stimulation improves cognitive and emotional symptoms in major depression, as measured by the Beck Depression Inventory (BDI), Hamilton Depression Rating Scale, and Cambridge Neuropsychological Test Automated Battery tests. Ahmadizadeh et al. reported reductions in PTSD symptoms, anxiety, and depression using bilateral DLPFC stimulation, employing clinical scales such as the Diagnostic and Statistical Manual of Mental Disorders 5th edition, BDI, and Beck Anxiety Inventory (Ahmadizadeh et al., 2019). More recently, Van’t Wout-Frank et al. showed that combining tDCS with virtual reality (VR) exposure therapy enhanced PTSD symptom reduction compared to VR with sham stimulation, although VR alone did not produce substantial improvements (Van’t Wout-Frank et al., 2024).

Taken together, these studies highlight the versatility of tDCS across cognitive and emotional domains and suggest that its effects may vary according to stimulation site, population, and measurement tool. The emotional neutrality or mild protective effect observed in our study aligns with previous research demonstrating good emotional tolerability of tDCS in healthy participants. The small magnitude of mood change in our sample may reflect the relatively healthy, non-clinical population and the use of PANAS, which captures short-term emotional fluctuations. Accumulating evidence indicates that the cerebellum contributes to nonmotor functions, including emotion regulation and affective processing, via cerebello-thalamo-cortical circuits linking the cerebellum with prefrontal and limbic regions (Pierce & Péron, 2020; Rudolph et al., 2023). Non-invasive stimulation studies further show that cerebellar tDCS can modulate activity within prefrontal networks implicated in emotional processing, consistent with indirect effects on affect through distributed frontal–cerebellar pathways (Panico et al., 2024; Van Overwalle et al., 2024). In the present study, cerebellar stimulation produced modest changes in emotional affect that were comparable in direction to those observed following FEF stimulation, aligning with the view that cerebellar contributions to affect are integrated within broader cognitive–affective networks.

Despite these promising findings, several limitations warrant consideration. The modest sample size may limit generalizability, and the subjective nature of PANAS increases susceptibility to external influences, including participant expectations, daily stressors, and individual differences in affective expressiveness. Although blinding was implemented, placebo-like effects cannot be excluded, and baseline emotional variability may have contributed to observed fluctuations. Future research should include larger, more diverse samples, incorporate objective physiological or behavioral measures, and systematically examine dose–response relationships across stimulation parameters.

Importantly, this study examined only the acute effects of a single 25 min anodal tDCS session, without repeated-session or longitudinal assessments. Accordingly, the findings cannot address cumulative or long-term effects. Consistent with the primary analyses, no significant main effects of stimulation type or site were observed for either positive or negative affect, and the small descriptive reductions in PANAS scores should be interpreted cautiously. Multi-session and longitudinal designs will be necessary to determine whether repeated tDCS produces additive or sustained effects on emotional affect.

To isolate the effect of stimulation location, identical tDCS parameters were applied across FEF and cerebellar conditions, minimizing confounds related to current intensity, duration, and electrode configuration. While this approach facilitates direct comparison, future studies may benefit from site-specific parameter optimization to account for regional differences in cellular architecture, conductivity, and network connectivity that may influence responsiveness. In this context, the use of a 2.0 mA stimulation intensity—commonly employed in prior research and shown to produce reliable neuromodulatory effects within established safety guidelines (Knechtel et al., 2013; Lee & Yoo, 2024)—provides consistency but does not capture potential dose-related effects. Accumulating evidence indicates that tDCS outcomes are dose-dependent and non-linear, with substantial interindividual variability and potentially distinct sensitivity profiles across behavioral and affective domains (Hsu et al., 2021; Perrey, 2024). Because intensity was not manipulated, lower or individualized stimulation levels may differentially influence affect, warranting systematic investigation in future work.

The implications of these findings extend beyond affective neuroscience. In ophthalmology, tDCS is gaining interest as a potential adjunctive treatment for conditions such as amblyopia, where cortical suppression plays a central role, and convergence insufficiency, where targeted neuromodulation may enhance therapeutic outcomes. Our results suggest that tDCS is emotionally safe in healthy participants, supporting its potential integration into visual rehabilitation or other non-psychiatric interventions. Understanding emotional responses to neuromodulation is essential for ensuring patient comfort, adherence, and holistic treatment evaluation.

## 5. Conclusions

This study demonstrates that anodal tDCS targeting the FEF and cerebellum is emotionally safe and may confer modest protective effects against mood decline. Compared with sham stimulation, anodal stimulation produced a smaller reduction in positive affect and a greater reduction in negative affect. These results underscore the importance of monitoring emotional outcomes in neuromodulation studies and support continued investigation of tDCS in both ophthalmic and affective neuroscience applications. Future work incorporating larger samples, multimodal emotion assessments, and clinical populations may further clarify the therapeutic potential of tDCS.

## Figures and Tables

**Table 1:** Summary statistics for each category, including mean, standard deviation, median, range, skewness, and kurtosis.

	Mean	Std	Median	Range	Skewness	Kurtosis
Cumulative sum of Positive PANAS Score Before Anodal tDCS in all participants	28.62	9.17	27	40	0.24	−0.23
Cumulative sum of Positive PANAS Score After Anodal tDCS in all participants	25.95	9.01	26	40	0.48	0.17
Cumulative sum of Positive PANAS Score Before Sham tDCS in all participants	29.78	8.03	31	38	0.17	0.16
Cumulative sum of Positive PANAS Score After Sham tDCS in all participants	25.68	9.92	25	47	0.25	0.28
Cumulative sum of Negative PANAS Score Before Anodal tDCS in all participants	13.68	4.57	13	22	2.04	5.25
Cumulative sum of Negative PANAS Score After Anodal tDCS in all participants	12.48	4.17	11	20	2.55	7.24
Cumulative sum of Negative PANAS Score Before Sham tDCS in all participants	14.8	5.38	13	20	1.39	1.4
Cumulative sum of Negative PANAS Score After Sham tDCS in all participants	12.95	4.21	11	20	2.22	6.01
FEF Group Cumulative sum of Positive PANAS Score Before Anodal tDCS in all participants	26.38	8.39	26.5	40	0.63	1.41
FEF Group Cumulative sum of Positive PANAS Score After Anodal tDCS in all participants	24.65	8.31	24.5	38	1	2.02
FEF Group Cumulative sum of Positive PANAS Score Before Sham tDCS in all participants	29.04	8.18	30	38	0.277	0.59
FEF Group Cumulative sum of Positive PANAS Score After Sham tDCS in all participants	24.48	10.23	22	47	0.33	0.55
FEF Group Cumulative sum of Negative PANAS Score Before Anodal tDCS in all participants	13.69	4.16	12	14	1.15	0.18
FEF Group Cumulative sum of Negative PANAS Score After Anodal tDCS in all participants	12	3.48	11	13	2.2	4.02
FEF Group Cumulative sum of Negative PANAS Score Before Sham tDCS in all participants	13.8	5.13	11	19	1.75	2.72
FEF Group Cumulative sum of Negative PANAS Score After Sham tDCS in all participants	13.08	4.64	11	20	2.35	6.6
Cerebellum Group Cumulative sum of Positive PANAS Score Before Anodal tDCS in all participants	32.05	9.5	33	33	−0.37	−0.48
Cerebellum Group Cumulative sum of Positive PANAS Score After Anodal tDCS in all participants	27.94	9.92	29	36	−0.12	−0.49
Cerebellum Group Cumulative sum of Positive PANAS Score Before Sham tDCS in all participants	30.93	7.92	31.5	29	0.05	0.03
Cerebellum Group Cumulative sum of Positive PANAS Score After Sham tDCS in all participants	27.56	9.44	28.5	37	0.26	0.42
Cerebellum Group Cumulative sum of Negative PANAS Score Before Anodal tDCS in all participants	13.7	5.26	13	22	2.85	9.78
Cerebellum Group Cumulative sum of Negative PANAS Score After Anodal tDCS in all participants	13.23	5.06	12	20	2.6	7.64
Cerebellum Group Cumulative sum of Negative PANAS Score Before Sham tDCS in all participants	16.37	5.56	15.5	20	1.2	1.32
Cerebellum Group Cumulative sum of Negative PANAS Score After Sham tDCS in all participants	12.75	3.58	11.5	13	1.75	3.43

## Data Availability

Data supporting reported results can be found in the Supplementary Materials section.

## Supplementary Materials

The following supporting information can be downloaded at: https://www.mdpi.com/article/10.3390/bs16060897/s1.

## Abbreviations

The following abbreviations are used in this manuscript:

FEF	Frontal Eye Field
PANAS	Positive and Negative Affect Schedule
tDCS	Transcranial direct current stimulation
PTSD	Post-Traumatic Stress Disorder
TMS	Transcranial magnetic stimulation
VR	Virtual reality
BDI	Beck Depression Inventory
POMS	Profile of mood states
